# Influence of Dietary Supplementation of Propolis and Bee Pollen on Liver Pathology in Broiler Chickens

**DOI:** 10.3390/ani8040054

**Published:** 2018-04-09

**Authors:** Ivana Klaric, Mirela Pavic, Ivan Miskulin, Valerija Blazicevic, Albina Dumic, Maja Miskulin

**Affiliations:** 1 Faculty of Agriculture in Osijek, Josip Juraj Strossmayer University of Osijek, 31 000 Osijek, Croatia; ivana.klaric@pfos.hr; 2 Faculty of Veterinary Medicine, University of Zagreb, 10 000 Zagreb, Croatia; mirela.pavic@vef.hr; 3 Faculty of Medicine Osijek, Josip Juraj Strossmayer University of Osijek, 31 000 Osijek, Croatia; ivan.miskulin@mefos.hr (I.M.); valerija.blazicevic@mefos.hr (V.B.); albina.dumic@gmail.com (A.D.)

**Keywords:** animal feeding, supplementation, broilers, histopathology, liver, propolis, bee pollen

## Abstract

**Simple Summary:**

Today, there are increased demands for consumers to use natural products as alternative additives in broiler feeding. In this study, we evaluated the effects of propolis and bee pollen as potential new additives on liver pathology in broilers. The results of this study showed that supplementation of broilers with propolis and/or bee pollen has a strong protective effect on liver pathology. Thus, these natural agents can be used as alternative additives in modern broiler production. Such an approach will enable the production of chicken meat enriched with bioactive substances from propolis and/or bee pollen, such as flavonoids, that have been proven beneficial for human health.

**Abstract:**

One of the major problems in intensive breeding of chickens is liver damage. The objective of this study was to determine the influence of dietary supplementation with propolis and bee pollen on liver pathology in broiler chickens. The study was conducted on 200 Ross 308 chickens equally distributed by sex that were divided into five groups. Throughout the whole study, the control group of chickens was fed with a basal diet, while the experimental groups of chickens were fed with the same diet further supplemented with propolis and bee pollen, each supplement given separately or in combination in a certain proportion. The study showed that the clusters of lymphocytes in the hepatocytes, the vacuolar degeneration and necrosis of the liver parenchyma, the bile ductule hyperplasia, and the various forms of pathological changes in the liver arteries and veins were more frequent in liver tissue samples of the control group compared to liver tissue samples of all the experimental groups (*p* < 0.001). The study further showed that all the previously mentioned histopathological lesions of liver tissue were always more extensive in the liver tissue samples of the control group than in the liver tissue samples of all the experimental groups (*p* < 0.001). The supplementation of broiler chickens with propolis and/or bee pollen has a strong protective effect on liver pathology in broiler chickens.

## 1. Introduction

Propolis is a natural resinous bee product [1]. It is more than 50% composed of lipophilic substances such as leaves, plant resins and balsams, plant latex, and vegetable glue. The other components are waxes (30%), essential and aromatic oils (10%), and pollen (5%). The rest of it (5%) is a mixture of different substances such as polyphenolic substances (e.g., flavonoids, organic phenols, ketones, and terpenes) and organic debris (i.e., wood fragments) [2]. Moreover, propolis contains minerals (such as Mg, Ca, K, Na, Cu, Zn, Mn, and Fe), vitamins (such as B1, B2, B6, C, and E), fatty acids, and some enzymes [3].

Bee pollen consists of the male gametophytes of seed plants [4,5]. To date, about 250 chemical compounds of great variety have been found in pollen including carbohydrates, fats, proteins, vitamins, macroelements, microelements, antibiotics (i.e., inhibins), hormones, enzymes, organic acids, essential oils, rutin, etc. [4]. Bees collect pollen from flowers and mix it with their own digestive enzymes. Bee pollen is rich in proteins (comprising 25% of it) and essential amino acids. Moreover, pollen is also 6% comprised of oils and 51% comprised of polyunsaturated fatty acids. The main polyunsaturated fatty acids are linolenic acid (39%), palmitic acid (20%) and linoleic acid (13%). Bee pollen contains more than 12 vitamins (i.e., B-complex vitamins, vitamins A, C, D, E, and K3), 28 minerals, 11 enzymes or coenzymes, and 11 different carbohydrates, and these comprise 35–61% of pollen. The carbohydrates are mainly glucose and fructose [6].

The biological activity of propolis and bee pollen depends on the active substances of the polyphenolic fraction. This is consisted mainly of flavonoids but also contains aromatic acids, triterpenes, lignans, carotenoids, phytosterols, polyphenols etc. [7,8]. These bioactive components of propolis and bee pollen are responsible for the antibacterial, antiviral, antifungal, antiprotozoal, antimicrobial, analgesic, anti-inflammatory, antioxidant, locally anesthetic, cytostatic, i.e., anticancer, and immune-stimulating and immunomodulatory effects of these substances in humans and animals [4,9,10,11].

There is scarce information about the effects of propolis and bee pollen on the pathology of individual organs and tissues, such as liver, that are predisposed to damage as a result of the intensive breeding of poultry [12]. The liver is a vital organ that plays a key role in the detoxification of exogenous and endogenous substances. It also performs a wide range of metabolic activities required for homeostasis, nutrition, and immune defense [13]. Subclinical infections are a major problem in broiler breeding and also result in liver damage [14]. Several studies have shown that subclinical infections with *Clostridium perfringens* are one of the main causes of liver damage in broiler chickens [15,16]. Histopathologically, massive proliferation of bile ductules, proliferation of connective tissue around the ductules, hepatocyte necrosis, and massive lymphocyte and heterophil infiltration are observed in liver tissue as a result of its damage [14,15,16]. Additionally, in liver tissue of fast-growing broiler chickens, a significant group of degenerative regressive lesions have also been found [14]. The cause of degenerative changes in fast-growing broiler chickens is a prolonged state of hypoxemia leading to hypoxia [14,17]. Under conditions of continuous and high demand for oxygen and nutrients, the liver tissue may respond with regressive lesions (i.e., parenchymatous, vacuolar, and fatty degeneration, steatosis, and necrosis of hepatocytes) [14].

The objective of this study is to evaluate the influence of dietary supplementation of propolis and bee pollen on liver pathology in broiler chickens.

## 2. Materials and Methods 

### 2.1. Animals and Diets

A total of 200 day-old un-sexed Ross 308 chickens were used in the present study. The feeding trial of the chickens was carried out on a farm in eastern Croatia under the supervision of the Department of Animal Husbandry, Faculty of Agriculture in Osijek, Josip Juraj Strossmayer University of Osijek. The experimental protocol was approved by the Committee for Animal Welfare of the Faculty of Agriculture in Osijek, Josip Juraj Strossmayer University of Osijek (Approval code: 2158-94-02-18-01).

Chickens were divided into five groups and randomly allocated to five floor pens containing fresh wood shavings to the depth of 10 cm in an environmentally controlled house. The experiment was a completely randomized design divided into five dietary treatments with two replicate groups of 20 birds per pen (5 diets × 2 replicates). The groups of chickens were housed under the same conditions during the whole experimental period. Temperature, humidity, and lighting in the facility were maintained within optimum limits, according to the manufacturer’s recommendations for the Ross 308 hybrid [18]. Breeding was conducted on wooden sawdust and lasted for six weeks (42 days). During the study, feed and water were offered to chickens’ *ad libitum*. In the experiment there were a control group (K) and four experimental groups (P1, P2, P3, P4). For ensuring an effective monitoring of all the investigated indicators, all the chickens were marked with a leg ring on the seventh day of the trial.

From days 1–21 of the study, chickens were fed a mixture of broiler starter. From days 22–42 of the study, chickens were fed a mixture of broiler finisher. The composition and calculated analyses of feed mixtures used in the chicken feeding are shown in Table 1. Throughout the study the control group (K) of chickens was fed a standard diet without additives, while the experimental groups of chickens (P1, P2, P3, and P4) were fed the same diet further supplemented with propolis and/or bee pollen: P1 group was given diet with 0.25 g of propolis per kilogram of diet and 20 g of bee pollen per kilogram of diet; the P2 group was given diet with 0.5 g of propolis per kilogram of diet; the P3 group was given diet with 1.0 g of propolis per kilogram of diet; and the P4 group was given diet with 20 g of bee pollen per kilogram of diet. Inclusion of propolis and bee pollen into the feed mixture was performed using a vertical mixer (Briketstroj Ltd., Valpovo, Croatia).

### 2.2. Sample Collection and Analysis

At the end of the feeding period (i.e., day 42), 10 birds from each group were selected at random and slaughtered for a necropsy examination. Fifty liver samples (10 from each group) were removed from the birds directly after slaughtering and fixed in 10% neutralized formalin. After that, fixed tissue samples were transported to the Department of Pathology and Forensic Medicine of the Faculty of Medicine in Osijek, where they were further processed. The tissues were then dehydrated with increasing concentrations of ethyl alcohol (70, 90, 96, and 100%), cleared in xylene, and embedded in paraffin. The 5 μm-thick microtome sections were stained with hematoxylin and eosin and assessed under a light microscope (Olympus CX40, Olympus Optical Co. Ltd., Hamburg, Germany).

In each examined sample of liver tissue, the following pathological patterns were observed and recorded: the existence of clusters of lymphocytes among the hepatocytes; the existence of different forms of regressive lesions of hepatocytes (such as degeneration, vacuolar degeneration, steatosis, and necrosis of the liver parenchyma) and their extent; the existence of bile ductule hyperplasia; the existence of pathological changes in the liver arteries (such as arterial hyperplasia, fibromuscular arterial dysplasia, and the induration of the arterial wall) and their extent; and the existence of pathological changes in the liver veins (such as thickening of the walls of veins and hyperplasia of the fibrous tissue within the walls of the veins) and their extent. The extensiveness of histopathological lesions in the chickens’ livers were described with labels of “does not exist,” “extremely poorly expressed,” “poorly expressed,” “moderately expressed,” “strongly expressed,” or “extremely strongly expressed.”

### 2.3. Statistical Analysis

Upon confirming the normality of data distribution using the Shapiro-Wilkinson test, all data were processed using methods of descriptive statistics. The categorical variables were described in absolute and relative frequencies. Fisher’s exact test was used for the comparison of categorical variables between the groups. The level of statistical significance was set at *p* < 0.05. Statistical analysis was done using the statistical package Statistica for Windows 2010 (version 10.0, StatSoft Inc., Tulsa, OK, USA).

## 3. Results

**Figure 1:**
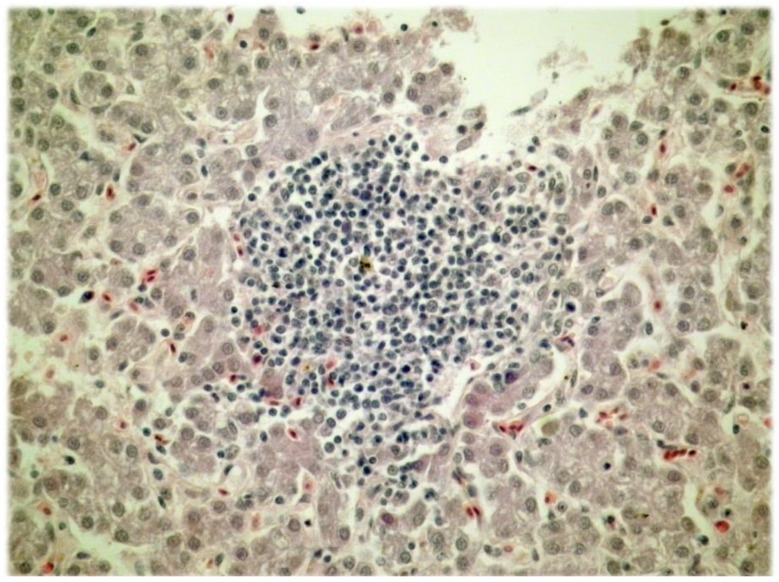
The cluster of lymphocytes among the hepatocytes (H&E; ×400).

The histological analysis of liver tissue samples revealed that the clusters of lymphocytes among the hepatocytes were more frequent in liver tissue samples from the control group compared to liver tissue samples of all the experimental groups (*p* < 0.001; Fisher’s exact test). The typical cluster of lymphocytes among hepatocytes is shown in Figure 1.

**Figure 2:**
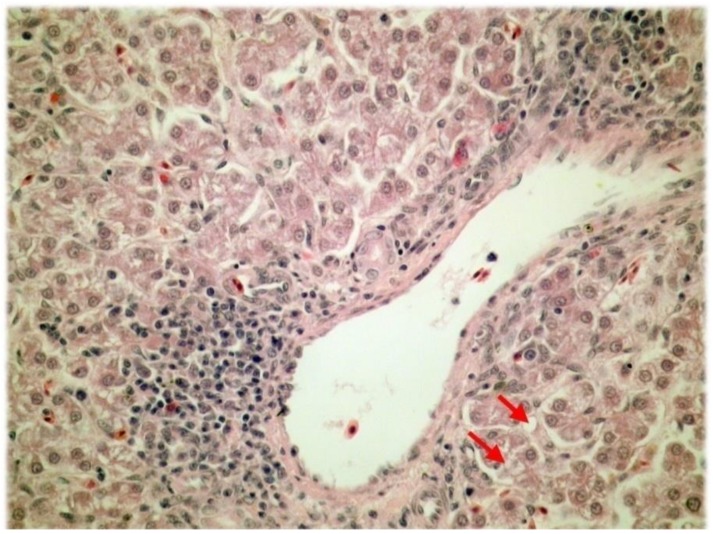
The vacuolar degeneration of the liver parenchyma (H&E; ×400).

The histological analysis of liver tissue samples further revealed that vacuolar degeneration and necrosis of the liver parenchyma were more frequent and in greater extent in liver tissue samples of the control group (*p* < 0.001; Fisher’s exact test) (Table 2). The vacuolar degeneration of the liver parenchyma is shown in Figure 2.

Regarding the extensiveness of different forms of regressive lesions of hepatocytes, the histological analysis showed that the vacuolar degeneration of the liver parenchyma, the steatosis of the liver parenchyma, and the necrosis of the liver parenchyma were more extensive in liver tissue samples of the control group than in liver tissue samples of all the experimental groups (*p* < 0.001, *p* = 0.002, *p* < 0.001, respectively; Fisher’s exact test).

**Figure 3:**
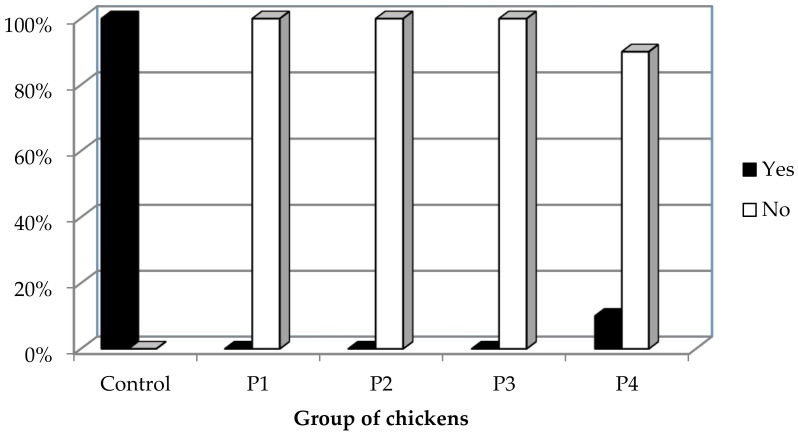
The presence of bile ductule hyperplasia in the chickens’ liver on the 42nd day of the feeding period (Fisher’s exact test; *p* < 0.001).

The histological analysis of liver tissue samples revealed that bile ductule hyperplasia was more frequent in liver tissue samples of the control group compared to liver tissue samples of all the experimental groups (*p* < 0.001; Fisher’s exact test) (Figure 3).

The histological analysis of liver tissue samples showed that arterial hyperplasia, fibromuscular arterial dysplasia, and the induration of the arterial wall were more frequent in liver tissue samples of the control group compared to liver tissue samples of all the experimental groups (*p* < 0.001; Fisher’s exact test).

Regarding the extensiveness of different forms of pathological changes in the liver arteries, the histological analysis further showed that arterial hyperplasia, fibromuscular arterial dysplasia, and the induration of the arterial wall were more extensive in liver tissue samples of the control group of chickens than in the liver tissue samples of all the experimental groups of chickens (*p* < 0.001; Fisher’s exact test).

The histological analysis of liver tissue samples revealed that the thickening of the walls of veins and the hyperplasia of the fibrous tissue within the walls of veins were more frequent in liver tissue samples of the control group compared to liver tissue samples of all the experimental groups *(p <* 0.001; Fisher’s exact test) (Table 3).

Regarding the extensiveness of different forms of pathological changes in the liver veins, the histological analysis further revealed that thickening of the walls of veins and the hyperplasia of the fibrous tissue within the walls of veins were more extensive in liver tissue samples of the control group than in the liver tissue samples of all the experimental groups (*p* < 0.001; Fisher’s exact test).

## 4. Discussion

Considering the results of the histological analysis of the liver of broiler chickens on the 42nd day of the feeding period, this study showed that that the clusters of lymphocytes among the hepatocytes, the vacuolar degeneration and necrosis of the liver parenchyma, the bile ductule hyperplasia, and various forms of pathological changes in the liver arteries and veins were more frequent in liver tissue samples of the control group compared to samples of all the experimental groups (*p* < 0.001). The study further showed that the all the aforementioned histopathological lesions of the liver tissue were more extensive in the liver tissue of the control group than in the liver tissue of the experimental groups (*p* < 0.001). Considering the clusters of lymphocytes, it is necessary to emphasize that inflammation in the absence of infection in the liver is often initiated when damaged hepatocytes, undergoing cell death, expose intracellular molecules that can be recognized by cells of the innate immune system. In particular, resident macrophages or Kupffer cells become activated and trigger an inflammatory response through common pathways involving the NLRP3 inflammasome and interleukin-1 beta (IL-1b) [19]. Studies have shown that propolis extract has an ability to inhibit NLRP3 activation through various mechanisms among all through the decrease of the reactive oxygen species production, and impair of interleukin-1 beta expression. [20,21].

The results of our study, in which bee pollen supplementation of 20 g/kg had been used solely or in combination with propolis, are in contrast with the results of the study by Babinska et al. (2012) in which there were control group and groups supplemented with 0.25 g/kg of propolis, 5 g/kg of bee pollen and 0.25 g/kg of propolis and 5 g/kg of bee pollen, respectively [4]. In their study, the most intensive and the highest number of regressive lesions of the liver were found in the liver tissue of chickens that consumed a diet supplemented either with bee pollen or with the combination of propolis and bee pollen [4]. Furthermore, their study found that bile ductule hyperplasia occurred often in chickens of all groups, with the most extensive lesions being in the livers of broilers that consumed the diet supplemented with bee pollen and the livers of broilers that consumed the diet supplemented with propolis [4]. However, they did not find statistically significant differences between the control group of chickens and the experimental groups of chickens with regards to the existence of clusters of lymphocytes among the hepatocytes [4]. Considering the various forms of pathological changes in the liver arteries, this study showed that these histopathological lesions were more frequent in the livers of broilers that consumed a diet supplemented with the combination of propolis and bee pollen [4]. The latter finding can be explained by the fact that amount of propolis in group of chickens that consumed a diet supplemented with the combination of propolis and bee pollen is much lower than the amount of propolis in the group that consumed the mixture supplemented only with propolis.

The results of our study are largely consistent with that of Babinska et al. (2013), in which there were control group and groups supplemented with 0.01 or 0.05 g/kg of propolis [12]. This study demonstrated that in the livers of broilers fed diets supplemented with propolis, there were no regressive lesions of hepatocytes; those lesions were most extensively expressed in the livers of broilers in the control group. This study also found that bile ductule hyperplasia was extensively expressed in the livers of broilers in the control group. Finally, this study showed that various forms of pathological changes in the liver arteries and liver veins were most commonly found and were most extensively expressed in the livers of broilers in the control group [12].

The studies dealing with the influence of propolis and/or bee pollen supplementation on the liver pathology have shown that the addition of these substances into the broiler diet protected liver tissue against the adverse effects of various hepatotoxic factors [22,23,24,25]. These factors often lead to the formation of various types of regressive lesions such as degeneration, vacuolar degeneration, steatosis, and necrosis of the liver parenchyma [22,23,24,25]. This property of propolis has been attributed to its phenolic components (including flavonoids) that are intense antioxidants and ensure protection against lipid oxidation in the cell membrane [4,25,26].

In their studies in rats, Nirala and Bhadauria (2008) and Bhadauria and Nirala (2009) showed the protective effect of alcoholic extract of propolis against regressive lesions in the liver and kidneys induced by paracetamol [24,26]. Bhadauria et al. (2008) further found a positive effect of propolis against liver damage in rats caused by carbon tetrachloride [27]. El-Mahalaway et al. (2015) demonstrated a potential protective effect of propolis against experimentally induced hepatotoxicity (via D-galactosamine (D-GalN)) and lipopolysaccharide (LPS) induced hepatitis in rats. The observed hepatoprotective effect of propolis was attributed to its strong antioxidant, anti-inflammatory, and antiapoptotic activities [13].

Regarding the influence of propolis and/or bee pollen on the pathology of liver arteries and veins, present study indicated that there are beneficial effects of supplementation with these bee products on the pathological structures of blood vessels. These effects are attributed to the strong antioxidant properties of such products [12,25]. Nader et al. (2010) demonstrated an inhibitory effect of propolis on the development of atherosclerosis in rabbits fed with a diet enriched with cholesterol. The observed effect was attributed to the strong antioxidant properties of propolis [28]. The described positive effect of propolis on blood vessels is particularly important, bearing in mind that fast growing broilers quickly develop pathological lesions in their arteries. This can then lead to the thickening of their walls because of the hyperplasia and/or hypertrophy of smooth muscles or because of the hyperplasia of fibrous tissue, and this can result in a significant narrowing or complete occlusion of the vascular lumen as a result of fibromuscular dysplasia [29].

Present study revealed significant findings regarding the results achieved by the different protocols of supplementation. It was shown that groups of chicken that were fed mixtures supplemented only with propolis in high amounts (P2 and P3) displayed better results in comparison to the control group but also to the group that was fed mixture supplemented only with bee pollen (P4) and the group (P1) that was fed mixture supplemented with the combination of propolis and bee pollen. These results can be explained by the quantity of propolis with the proven anti-inflammatory activities which through the reduction of the secretion of interleukin-1 beta inhibits the inflammasome activation [21].

However, this study is not without limitations. Due to limited accommodation possibilities and the justifiable wish to minimize the number of animals used in this study, the replicates were individual birds within the pens, although the replication by pen is standard in poultry research. Considering the tested feed additives and main goal of this study we believe that described design of the study did not affect the results.

## 5. Conclusions

In conclusion, this study demonstrated that dietary supplementation with propolis and/or bee pollen has strong protective effects on the liver. It has been shown that propolis and bee pollen successfully protect liver tissue from various forms of regressive liver lesions, such as degeneration, vacuolar degeneration, steatosis, and necrosis of the liver parenchyma, as well as from other pathological findings in the liver tissue, such as the clusters of lymphocytes among the hepatocytes and bile ductule hyperplasia. Finally, this study clearly showed that propolis and bee pollen also have strong beneficial effects on the pathological structures of liver arteries and veins.

## Figures and Tables

**Table 1:** The composition and calculated analysis of feed mixtures used in chicken feeding.

**Fodders (%)**	**Starter**	**Finisher**
	**1–21 day**	**22–42 day**
Corn grain	45.00	46.10
Flour middling	2.80	3.00
Dehydrated alfalfa	2.80	4.00
Soybean meal	20.20	10.00
Sunflower meal	4.00	4.00
Yeast	4.00	3.00
Full fat soybean	12.40	20.00
Vegetable oil	3.70	5.00
Monocalcium phosphate	1.20	1.20
Limestone	1.60	1.40
Salt	0.30	0.30
Premix *	1.00	1.00
Pigozen 801	1.00	1.00
Total	100.00	100.00
**Calculated Analysis**		
Crude protein (%)	21.02	19.15
Crude fat (%)	8.36	10.96
Crude fiber (%)	4.96	5.05
Lysine (%)	1.11	0.96
Methionine (%)	0.66	0.61
Tryptophan (%)	0.26	0.23
Calcium (%)	1.04	0.98
Phosphorous (%)	0.70	0.67
ME (Metabolisable energy, MJ/kg)	12.30	13.10

* Each kilogram of premix contains 1,200,000 IU of vitamin A; 200,000 IU of vitamin D3; 3000 mg of vitamin E; 250 mg of vitamin K3; 150 mg of vitamin B1; 600 mg of vitamin B2; 200 mg of vitamin B6; 1 mg of vitamin B12; 50 mg of folic acid; 4400 mg of niacin; 1500 mg of Ca panthothenate; 10 mg of biotin; 50,000 mg of choline chloride; 5000 mg of iron; 700 mg of copper; 8000 mg of manganese; 5000 mg of zinc; 75 mg of iodine; 20 mg of cobalt; 750 mg of magnesium; 15 mg of selenium; 10,000 mg of antioxidant BHT; 100,000 mg of methionine; and 1000 g of herbal carrier.

**Table 2:** The presence of different forms of regressive lesions of hepatocytes in the chickens’ liver on the 42nd day of the feeding period.

The forms of regressive lesions of hepatocytes		Group of chickens					* *p*
		Control (K)	P1	P2	P3	P4	
Degeneration of the liver parenchyma	Yes %	90.0	60.0	70.0	60.0	60.0	0.507
	No %	10.0	40.0	30.0	40.0	40.0	
Vacuolar degeneration of the liver parenchyma	Yes %	90.0	10.0	20.0	0	0	<0.001
	No %	10.0	90.0	80.0	100.0	100.0	
Steatosis of the liver parenchyma	Yes %	90.0	90.0	60.0	60.0	70.0	0.319
	No %	10.0	10.0	40.0	40.0	30.0	
Necrosis of the liver parenchyma	Yes %	100.0	30.0	0	0	10.0	<0.001
	No %	0	70.0	100.0	100.0	90.0	

* Fisher’s exact test; K = control group; P1 = feed mixture + 0.25 g of propolis per kilogram of feed mixture + 20 g of bee pollen per kilogram of feed mixture; P2 = feed mixture + 0.5 g of propolis per kilogram of feed mixture; P3 = feed mixture + 1.0 g of propolis per kilogram of feed mixture; P4 = feed mixture + 20 g of bee pollen per kilogram of feed mixture.

**Table 3:** The presence of different forms of pathological changes in the chickens’ liver veins on the 42nd day of the feeding period.

The forms of pathological changes in the liver veins		Group of chicken					* *p*
		Control (K)	P1	P2	P3	P4	
Thickening of the walls of veins	Yes %	100.0	10.0	10.0	10.0	10.0	<0.001
	No %	0	90.0	90.0	90.0	90.0	
The hyperplasia of the fibrous tissue within the walls of the veins	Yes %	100.0	10.0	10.0	10.0	10.0	<0.001
	No %	0	90.0	90.0	90.0	90.0	

* Fisher’s exact test; K = control group; P1 = feed mixture + 0.25 g of propolis per kilogram of feed mixture + 20 g of bee pollen per kilogram of feed mixture; P2 = feed mixture + 0.5 g of propolis per kilogram of feed mixture; P3 = feed mixture + 1.0 g of propolis per kilogram of feed mixture; P4 = feed mixture + 20 g of bee pollen per kilogram of feed mixture.
